# Cerebral Hemodynamic Evaluation After Cerebral Recanalization Therapy for Acute Ischemic Stroke

**DOI:** 10.3389/fneur.2019.00719

**Published:** 2019-07-03

**Authors:** Zhe Zhang, Yuehua Pu, Donghua Mi, Liping Liu

**Affiliations:** Department of Neurology, Beijing Tiantan Hospital, Capital Medical University, Beijing, China

**Keywords:** recanalization, thrombolysis, mechanical thrombectomy, hemodynamics, hemodynamic evaluation, ischemic stroke, hyperperfusion

## Abstract

Cerebral recanalization therapy, either intravenous thrombolysis or mechanical thrombectomy, improves the outcomes in patients with acute ischemic stroke (AIS) by restoring the cerebral perfusion of the ischemic penumbra. Cerebral hemodynamic evaluation after recanalization therapy, can help identify patients with high risks of reperfusion-associated complications. Among the various hemodynamic modalities, magnetic resonance imaging (MRI), computed tomography perfusion, and transcranial Doppler sonography (TCD) are the most commonly used. Poststroke hypoperfusion is associated with infarct expansion, while hyperperfusion, which once was considered the hallmark of successful recanalization, is associated with hemorrhagic transformation. Either the hypo- or the hyperperfusion may result in poor clinical outcomes. Individual blood pressure target based on cerebral hemodynamic evaluation was crucial to improve the prognosis. This review summarizes literature on cerebral hemodynamic evaluation and management after recanalization therapy to guide clinical decision making.

## Introduction

Patients with acute ischemic stroke (AIS) can benefit from recanalization therapy, including intravenous thrombolysis and mechanical thrombectomy, after neuroradiological evaluation within a restricted time window (1–7). Recanalization aims to restore effective and constant cerebral perfusion, to minimize final infarct volume and improve functional outcome (8). However, not all patients benefit from the treatment because of cerebral hemodynamic abnormalities after ischemic stroke. Cerebral autoregulation impairment, residual stenosis of culprit arteries, systemic hyper- or hypotension contribute to the abnormalities (9), which may further lead to symptomatic hemorrhagic transformation or unfavorable functional outcome (10–15).

Meanwhile, it is still under debate on blood pressure management after recanalization. The *2018 Guidelines for the Early Management of Patients with Acute Ischemic Stroke* recommended blood pressure (BP) should be controlled at <180/105 mmHg (16). Some neurocritical care experts suggest the target systolic BP to be <140 mmHg and mean arterial pressure of >70 mmHg to decrease the incidence of hemorrhage and reperfusion injury (17). Given the fact that cerebral hemodynamic state varies among patients, a “one size fits all” blood pressure could not possibly maintain effective and stable cerebral perfusion in every single patient. Physicians should decide individual BP targets based on the specific cerebral hemodynamic condition evaluated by multiple modalities from each patient, but not in a wide interval, to reduce the risk of both cerebral ischemia and hemorrhagic transformation.

In this mini-review, we outline the methods commonly used for cerebral hemodynamic condition assessment. We focus on the most recent studies performed with various modalities in patients who received recanalization therapies. Moreover, we aim to provide an overview and reasonable expectation on the management of cerebral hemodynamic abnormalities.

## Overview of Modalities for Cerebral Hemodynamic Evaluation

Cerebral blood flow (CBF) is defined as the volume of blood moving through a determinate amount of brain tissue in a given time. It takes ~12% of cardiac output in healthy human beings (18). The absolute CBF is hard to measure, while some methods can be used to determine it indirectly, as well as other hemodynamic parameters (listed in Table 1). Among these methods, transcranial Doppler sonography (TCD), magnetic resonance imaging (MRI), and computed tomography (CT) are the most commonly used techniques (26).

**Table 1:** Cerebral hemodynamic parameters commonly used for ischemic stroke evaluation.

**Modality**	**Hemodynamic parameter**	**Definition of the hemodynamic parameter**	**Normal value (mean ± SD)**
Cerebral perfusion imaging (PET, SPECT, CTP, PWI, ASL)	CBF	The volume of blood moving through a determinate amount of brain tissue in a given time	Gray matter: 50 ± 15 ml/100 g/min White matter: 22 ± 5 ml/100 g/min^*^
	CBV	The volume of flowing blood for a given volume of brain (in units of milliliters of blood per 100 g of brain tissue)	Gray matter: 2.5 ± 0.4 ml/100 g White matter: 1.7 ± 0.4 ml/100 g^*^
	MTT	The mean amount of time it takes blood to pass through a given volume of brain (in units of seconds)	Gray matter: 4 s White matter: 4.8 s^*^
Cerebral blood flow sonography (TCD, TCCS)	Mean blood flow velocity	(Peak systolic velocity + [end diastolic velocity × 2])/3	MCA: 55 ± 12 cm/s ACA: 50 ± 11 cm/s PCA: 40 ± 10 cm/s BA: 41 ± 10 cm/s VA: 38 ± 10 cm/s (19)
Cortical oxygenation (PET, fMRI, NIRS)	OEF	1-(cerebral venous O_2_ content/cerebral arterial O_2_ content)	0.44 ± 0.06 (20)
	CMRO_2_	Cerebral arterial O_2_ content × OEF × CBF	3.3 ± 0.5 ml/100 ml/min (20)
	rSO_2_	Percentage of oxyhemoglobin of regional brain tissue	69 ± 6.0% (21)
Cerebral autoregulation	Mx	The Pearson correlation between CPP and MCA flow velocity over a 300 s moving window (22)	0.21 ± 0.16 (23)
	Phase shift (in low frequency range)	The displacement of a waveform relative to another waveform with the same period (24)	37.1 ± 3.0° (25)
	Gain (in low frequency range)	The damping effect between the input and output of the transfer function (24)	0.95 ± 0.08 cm/s/mmHg (25)

*SD indicates standard deviation; PET, positron emission tomography; SPECT, single-photon emission computed tomography; CTP, CT perfusion; PWI, perfusion weighed imaging; ASL, arterial spin labeling; CBF, cerebral blood flow; CBV, cerebral blood volume; MTT, mean transit time; TCD, transcranial Doppler sonography; TCCS, transcranial color-coded duplex sonography; MCA, middle cerebral artery; ACA, anterior cerebral artery; PCA, posterior cerebral artery; BA, basilar artery; VA, vertebral artery; fMRI, functional magnetic resonance imaging; NIRS, near-infrared spectroscopy; OEF, oxygen extraction fraction; CMRO_2_, cerebral metabolic rate of oxygen; rSO_2_, regional saturation of oxygen; Mx, mean flow index*.

* *Cited from https://radiopaedia.org/articles/*.

### Cerebral Blood Flow Sonography

TCD can measure the blood flow velocity (cm/s) of the major intracranial arteries, which consist of the circle of Willis (distal internal carotid arteries [ICA], middle cerebral arteries [MCAs], proximal anterior cerebral arteries [ACA], and posterior cerebral arteries [PCA]). Blood flow velocity correlates with CBF, making TCD an option for evaluating cerebral perfusion (27). TCD can also provide additional information such as collateral pathways and active microembolization (28).

TCD is low-cost and non-invasive and can be performed at bedside and continuously monitored, making it suitable for critically ill patients with a high risk of transfer. Therefore, evaluation with TCD prior to using other imaging approaches is reasonable, even though a cost-effectiveness analysis for various modalities for hemodynamic assessment after recanalization has not been conducted yet. However, velocity measured using TCD may also be influenced by residual stenosis, the insonation angle, and the operator's skill. Blood flow velocity cannot be obtained if a patient lacks an acoustic window.

### Cerebral Perfusion Imaging

(1) Nuclear medicine techniques include single-photon emission computed tomography (SPECT) and positron emission tomography (PET). SPECT uses an intravenous injection of a delivery compound labeled with the radioisotope technetium-99m (^99m^Tc), which can pass through the blood-brain barrier and be metabolized in cells. PET uses radioisotope-labeled water (${\text{H}}_{2}^{15}$O) as a radioactive diffusible contrast agent. Both SPECT and PET can be used to measure CBF and cerebral blood volume (CBV).

(2) MRI can determine CBF and visualize the perfusion territories of individual cerebral arteries by using either dynamic susceptibility contrast (DSC) or arterial spin labeling (ASL). DSC-MRI requires a bolus of gadolinium-based contrast injection for perfusion-weighted imaging. ASL uses magnetically labeled water as endogenous contrast agent in large arteries that perfuse the brain. The labeled arterial protons flow through the vascular tree and exchange water at the cerebral microcirculation, to generate a perfusion-weighed image (29).

(3) Perfusion CT (CTP) with rapid sequential scanning enables radiologists to draw time-concentration curves of the iodinated contrast bolus and then obtain the four parameters, CBV, mean transit time (MTT), time to peak (TTP), and CBF (Table 1).

ASL and CTP are usually harnessed to identify the infarct core and ischemic penumbra at the hyperacute stage of AIS (30). They are also suitable for detecting post-recanalization hyper- or hypoperfusion, especially for patients with a high risk of hemorrhagic transformation or early neurological deterioration if TCD cannot be performed. Nuclear medicine techniques (SPECT and PET) have severe limitations because of their complexity, invasiveness, and extra radiation exposure from the radiopharmaceutical besides CT during imaging, and thus are rarely used in clinical practice.

### Cerebral Oxygenation

1. PET can be used to measure the cerebral metabolic rate of oxygen (CMRO_2_) and oxygen extraction fraction (OEF) with rapid sequential scanning with ^15^O-labeled oxygen (^15^O_2_) and carbon dioxide (C^15^O_2_) (Table 1).
2. For functional (fMRI) and quantitative MRI (qMRI), radiologists use hypercapnic and hyperoxic respiratory challenges to calibrate blood oxygenation level-dependent (BOLD) fMRI signals to determine OEF and CMRO_2_ of the brain (31, 32). qMRI with T2′ mapping (1/T2′ = 1/T2^*^ – 1/T2) is an alternative radiological marker of cerebral OEF. Decreased T2′ values suggest focal hypoxia (33).
3. Near-infrared spectroscopy (NIRS) is as a non-invasive method to measure tissue concentrations of oxyhemoglobin and deoxyhemoglobin within the cerebral cortex through determining their optical difference, by placing probes on the patient's scalp. The probe emits near-infrared light capable of deep penetration through the bone to the cortex tissue (34), with the advantages of bedside application and continuous monitoring.

Combined with other perfusion imaging modalities, OEF and CMRO_2_ mappings are usually used for detecting ischemic penumbra when making therapeutic decisions. For patients receiving recanalization therapy, PET, fMRI, or qMRI are less commonly performed than NIRS because of challenges performing these techniques.

### Cerebral Autoregulation

CBF varies with cerebral perfusion pressure (CPP), which is the difference between the mean arterial and intracranial pressures (ABP and ICP, respectively), and is inversely associated with cerebrovascular resistance (CVR, mainly originating from the small pial arteries and precapillary arterioles). This relationship can be represented by the formula below.

(1) $$\begin{array}{r} \text{CBF}=\frac{ABP-ICP}{CVR} \end{array}$$

Cerebral autoregulation, the ability of cerebral vessels to maintain the CBF relatively constant over a wide range of systemic arterial blood pressure levels through numerous physiological mechanisms, may provide an association between CPP and CBF. To date there is no generally accepted “gold standard” capable of measuring cerebral autoregulation, yet researchers designed a variety of methods for the evaluation. These methods usually require continuous monitoring of the mean ABP (with finger photoplethysmography, for instance) and CBF (usually indirectly measuring the flow velocity with TCD) at the bedside. A simple method to quantitatively evaluate cerebral autoregulation is to calculate the correlation between the ABP and CBF (usually using MCA flow velocity instead) in a given time. The commonly used parameters include mean flow index (Mx), gain and phase shift (Table 1). A lower Mx, gain, and a larger phase shift suggest better cerebral autoregulation.

## Applications of Cerebral Hemodynamic Assessment After Recanalization

Most hemodynamic changes in the affected artery occur within the first 10 days after recanalization; hemodynamic deterioration is associated with clinical worsening and poor functional outcome (35). Prompt assessment after recanalization may aid in early warning of cerebrovascular complications.

### Transcranial Doppler Sonography

By using TCD, complete recanalization is defined as a difference of <30% between the mean blood flow velocity of the affected artery and that of the contralateral side, with similar waveform shapes and a rating of grade 5 in the thrombolysis in brain ischemia (TIBI) scale (36). After intravenous thrombolysis, patients who achieved complete recanalization at 24 h with a TIBI grade of 5 had a significantly smaller infarct expansion than those with TIBI grades of ≤4, whereas in those assessed using magnetic resonance angiography (MRA), the difference was not significant (37). This result suggests that patients with TIBI grades of ≤4 had a higher risk of infarct growth even if the occluded artery reappeared entirely on MRA.

For patients who receive carotid endarterectomy (CEA) or carotid artery stenting (CAS), hyperperfusion syndrome is defined as an increase in mean flow velocity (MFV) of ≥100% as compared with the velocity of the same artery by TCD at baseline (38), which is not suitable, however, for patients who receive urgent recanalization because data at baseline cannot be acquired. Kneihsl et al. (39) retrospectively reviewed TCD examinations performed within 24 h after successful mechanical thrombectomy in 123 patients with MCA occlusion. They calculated the mean blood flow velocity index (= velocity of the recanalized MCA divided by that of the contralateral artery, MBF velocity index). Of the 123 subjects, 18 with hemorrhagic transformation had a higher index than those without (1.32 vs. 1.02, *P* < 0.001). The increased MBF velocity index was associated with hemorrhagic transformation and poor clinical outcome at 90 days. This finding could indicate that elevated ipsilateral MCA MBF velocity by 30% might predispose to intracerebral hemorrhage in anterior circulation infarction after successful mechanical thrombectomy.

However, other studies have reported inconsistent findings. A study (40) in which blood flow was observed using transcranial color-coded duplex sonography (TCCS) included 31 patients who received mechanical thrombectomy. Within 7 days after recanalization, segmental blood flow acceleration in the affected arteries (>35–40% than the contralateral arteries) was observed in 27 patients; none of them showed clinical deterioration. After excluding residual stenosis and vasospasm, the authors speculated that the focal increase in blood flow velocity after thrombectomy might be a result of endothelial layer disruption or intimal injury.

### Arterial Spin Labeling

Using the ASL technique, Bivard et al. (41) prospectively studied the perfusion state of 100 patients within 24 h after the onset of AIS, of whom 47 were treated with thrombolysis. They reported that the patients treated with thrombolysis were more likely to have hyperperfusion (26 vs. 15, *P* = 0.05). In all the subjects, ASL hyperperfusion of the initially ischemic area was associated with improved early clinical improvement and 90-day functional outcome. Among the patients who had achieved major reperfusion (>80% reduction in the ischemic lesion at 24 h), those with poststroke hyperperfusion had larger lesion volumes and higher National Institute of Health Stroke Scale (NIHSS) score than those with hypoperfusion on ASL (22 ± 9 ml vs. 9 ± 4 ml and 14 ± 5 vs. 6 ± 3, respectively). Thus, the authors concluded that on the basis of hyperperfusion at 24 h after stroke, patients with better tissue salvage and clinical outcomes can be identified.

More studies consider hyperperfusion a harmful complication of recanalization. Yu et al. (42) analyzed 361 ASL maps from 221 consecutive patients with MCA stroke. They defined hyperperfusion as patchy areas with increased CBF on ASL maps, either within or adjacent to the infarct lesion, as compared with the homologous contralateral hemisphere by visual assessment. Quantitative CBF was retrospectively calculated. Their work highlighted a strong association between having hyperperfusion at any time point and hemorrhagic transformation (odds ratio [OR] = 3.5, 95% confidence interval [CI] 2.0–6.3, *P* < 0.001). The mean CBF volumes in hyperperfusion and contralateral regions within the leptomeningeal MCA territory were 72.4 ± 25.7 and 45.5 ± 18.2 ml/(100 g·min), respectively (*P* < 0.001). The mean CBF volumes in hyperperfusion and contralateral regions within the perforator MCA territory were 53.1 ± 18 and 31.3 ± 12 ml/(100 g·min), respectively (*P* < 0.001). Therefore, patients with hyperperfusion (70–80% increase in CBF volume as compared with the non-infarct side) should be considered at-risk for hemorrhagic transformation.

Yu et al. (43) further suggested that both hypoperfusion and hyperperfusion after recanalization could lead to bad outcome. To prove this hypothesis, they prospectively assessed the reperfusion state of 90 patients who had received thrombolysis or endovascular treatment by using ASL perfusion MRI performed within 24 h. They calculated the reperfusion scores (RPS) based on the Alberta Stroke Program Early CT Score (ASPECTS) template, by deducting 1 point from 10, where the hypoperfusion or hyperperfusion of a scored region was given a point deduction (defined as <25 and >75% of the normal CBF range, respectively). They finally proved that the RPS could predict a favorable outcome (modified Rankin Scale [mRS] score of 0–2] at 3 months (area under the curve 0.93), even more accurately than diffusion-weighed imaging.

### Quantitative MRI

Seiler et al. (44) observed 11 patients with AIS after ICA or MCA occlusion by using qMRI. After successful thrombectomy, the T2′ value significantly increased within the ischemic tissue (*P* = 0.008), which indicated fast and complete restitution of oxygen supply after reperfusion and microstructural tissue integrity.

### Single-Photon Emission Computed Tomography

With SPECT, Abumiya et al. (45) evaluated CBF in 35 patients with AIS 1 h after tissue-type plasminogen activator (t-PA) infusion to predict patient outcome. The authors defined hypoperfusion as a CBF decrease of ≥25% and hyperperfusion as an increase of ≥25% as compared with those in the contralateral region. Ten patients with hyperperfusion had a significant improvement in NIHSS score within the first 24 h (ΔNIHSS 7.0 ± 4.7 vs. 3.5 ± 5.0, *P* = 0.033), while hypoperfusion volume was related to high NIHSS scores at 24 h (*r* = 0.555, *P* < 0.001) and high mRS scores at 3 months (*r* = 0.634, *P* < 0.001). It is interesting that 7 of the 8 hemorrhagic transformations occurred within the hypoperfusion area. This study suggested that hypoperfusion with a CBF volume decreased by ≥25% after recanalization might result in permanent brain tissue injury. It is also notable that the definition of hyperperfusion in this study was not the same as those used in the studies mentioned above (39, 42, 43).

### Near-Infrared Spectroscopy

Ritzenthaler et al. (46) briefly reported regional brain oxygen saturation (rSO_2_) changes in 17 patients with ischemic stroke treated with mechanical thrombectomy, determined using NIRS placed on the foreheads. They found that at baseline, rSO_2_ correlated with hemodynamic parameters (Tmax and MTT) within the forehead regions. They also noticed that an interhemispheric rSO_2_ difference diminished after recanalization. However, the rSO_2_ changes were not associated with the clinical outcome because NIRS did not detect brain lesions except for the frontal areas.

### Cerebral Autoregulation

Cerebral autoregulation impairment after AIS is associated with an increased risk of hemorrhagic transformation and cerebral edema, two significant complications that often lead to poor functional outcome (13). Reinhard et al. (47) studied cerebral autoregulation in 16 subjects with AIS after t-PA thrombolysis and 82 subjects as controls. By using correlation coefficient and transfer function analyses, they calculated the Mx and phase shift. In the good outcome group (mRS score 0–2), the Mx and phase did not change in the bilateral MCAs, whereas Mx worsened on the affected sides and the phase decreased bilaterally in the poor outcome group. Moreover, cerebral autoregulation impairment lasted for at least 5 days in the patients with unsuccessful recanalization.

## Management for Cerebral Hemodynamic Disturbances

To date, no guideline or consensus has been established regarding the management of hemodynamic disturbances after recanalization. Given the harmful effects of either hypoperfusion or hyperperfusion, both of which are well-recognized, treating these hemodynamic abnormalities based on cerebral hemodynamic evaluation may be reasonable.

### Cerebral Hypoperfusion and Incomplete Recanalization

According to the literature reviewed (37, 43, 45), physicians should focus on patients who have received recanalization therapy with TIBI scores of ≤4, assessed using TCD or a decrease in CBF volume of ≥25% compared with the contralateral homologous region on perfusion imaging, such as ASL-MRI and CTP, to identify the patients with cerebral hypoperfusion.

Reduced perfusion impairs the clearance of emboli (48); therefore, aspirin plus clopidogrel as dual antiplatelet therapy may be considered after weighing the benefits and risks of hemorrhage (49). Induced hypertension, to achieve a 10–30% increase in mean arterial pressure, may be warranted. A randomized pilot trial of 15 patients with AIS and >20% diffusion-perfusion mismatch reported that induced hypertension decreased the hypoperfused volume from a mean of 132 to 58 ml (50). In a mouse model of AIS, induced hypertension increased CBF and oxygenation in the infarct core and ischemic penumbra (51). A meta-analysis suggested that a lying flat head position at 0–15° might remarkably increase the flow velocity in the acute infarct side compared to an upright head position at 30° (52). However, the head position did not affect the disability outcomes in patients with AIS in a randomized multicenter trial (HeadPoST) (53). Hemodilution by volume expansion and high-dose albumin administration is not recommended for AIS because of the lack of a beneficial effect (16).

### Cerebral Hyperperfusion

**Figure 1:**
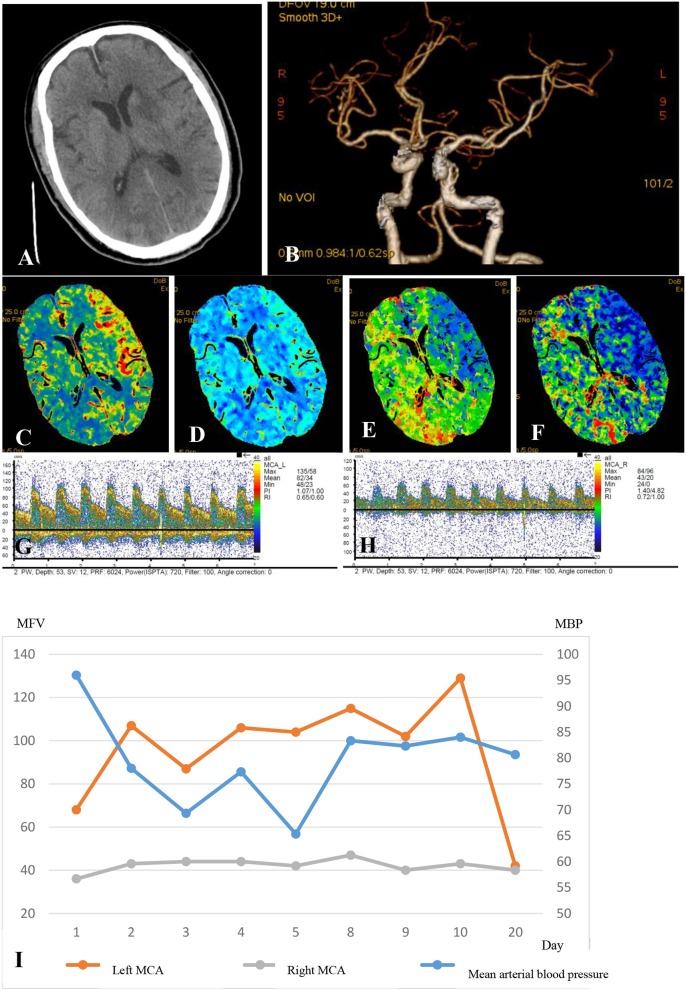
A 75-year-old man who received a successful mechanical thrombectomy in the C1 segment of the left ICA 3 h 50min after the onset of AIS. The NIHSS score at baseline was 22. Twenty-four hours after the recanalization, the NIHSS score slightly decreased to 20, and a multimodal hemodynamic evaluation was performed. The non-contrast CT scan shows the hypointense infarct core in the left perforating MCA territory **(A)**. The left ICA and MCA reappeared entirely on CTA, and the M1 segment of the left MCA showed no stenosis **(B)**. The CTP series showed markedly increased CBF **(C)**, mildly increased CBV **(D)**, significantly decreased MTT **(E)**, and higher TTP **(F)** in the left MCA territory compared to those in the infarct core. The mean flow velocity of the left MCA increased by 100% compared to that on the right side at the same depth (**G**, left MCA; **H**, right MCA). The patient was considered as having the hyperperfusion syndrome. The systolic blood pressure was controlled at <110 mmHg until the flow velocity of the left MCA was restored to the normal in 20 days **(I)**. The patient had no hemorrhagic transformation or epileptic seizures but had an mRS score of 5 at 90 days, although the final infarct volume was only 12ml. Electroencephalographic monitoring was also performed (data not shown).

Cerebral hyperperfusion had been long recognized as a hallmark of effective recanalization and favorable outcomes (54, 55). Although the safety of poststroke hyperperfusion is controversial, there are concerns that elevated BP and significantly increased CBF velocities may be associated with cerebral hyperperfusion syndrome, characterized by headaches, hemorrhagic transformation, and epileptic seizures (56). A possible mechanism is cerebral vessel wall injuries and vulnerability to the excessive blood flow due to the endovascular operation (Table 2, Figure 1) (57–59). We suggest that physicians pay attention to patients who have received recanalization therapy, particularly mechanical thrombectomy, with blood flow acceleration of >30–40% in the affected arteries compared to the blood flow velocity in the contralateral side determined using TCD, or a 70–80% increase in CBF volume compared with that on the non-infarct side determined by perfusion imaging (39, 40).

**Table 2:** Controversies regarding hyperperfusion after recanalization.

**References**	**Modality**	**Year**	**Number of subjects**	**Recanalization method**	**Definition of hyperperfusion**	**The time interval between recanalization and cerebral hemodynamic assessment**	**Impact of hyperperfusion**
Perren et al. (40)	TCCS	2017	31	Mechanical thrombectomy using Solitaire stent retriever	Focal PSV increase >35% compared to the contralateral homonym artery (MCA), >40% compared to the different depths of the same vessel (BA)	Within 7 days	Not harmful
Bivard et al. (41)	ASL	2012	100	Thrombolysis in 47 subjects	Not mentioned	Not mentioned. The time interval between the onset of stroke and ASL imaging was 24 h	Beneficial
Abumiya et al. (45)	SPECT	2013	35	Thrombolysis by a total dose of 0.6 mg/kg t-PA	A CBF increase ≥25% compared with the contralateral	1 h	Beneficial
Kneihsl et al. (39)	TCD	2017	123	Mechanical thrombectomy using stent retrievers or clot aspiration systems	Not defined. Mean MBF velocity index (= recanalized MCA MBF velocity/contralateral MCA MBF velocity) was 30% higher in ICH compared with non-ICH patients	6.6 ± 2.3 h (mean ± SD)	Harmful
Yu et al. (42)	ASL	2013	221	IV t-PA, IA t-PA, clot retrieval, and stenting in 102, 11, 41, and 8 subjects, respectively	Patchy areas with visually perceivable increased CBF on ASL maps compared with the homologous contralateral hemisphere	Not mentioned. The median time interval between the onset of stroke and initial ASL imaging was 7.05 h (IQR 3.35–18.23)	Harmful

*TCCS indicates transcranial color-coded duplex sonography; PSV, peak systolic velocity; MCA, middle cerebral artery; BA, basilar artery; ASL, arterial spin labeling; SPECT, single-photon emission computed tomography; t-PA, tissue plasminogen activator; CBF, cerebral blood flow; TCD, transcranial Doppler sonography; MBF, mean blood flow; SD, standard deviation; ICH, intracerebral hemorrhage; IV, intravenous; IA, intraarterial; IQR, interquartile range*.

Strict BP control is recommended as the primary management for hyperperfusion syndrome after CEA and CAS in patients with carotid atherosclerotic stenosis. Jørgensen et al. (60) observed that in patients with hyperperfusion syndrome after CEA, the median MFV of the affected MCA could be decreased from 92 to 56 cm/s without altering the MFV of the contralateral side by reducing the systemic mean ABP from 101 to 88 mmHg. In patients who have received CAS with a high risk of hyperperfusion syndrome and cerebral hemorrhage, comprehensive BP management (<120/80 mmHg as target BP) lowered the incidence of hyperperfusion and cerebral hemorrhage from 29.4 to 4.2% and from 17.6 to 0%, respectively (61).

Lower systolic BP seemed to be linearly associated with better clinical outcome in patients treated with recanalization (62, 63). However, the strength of antihypertensive treatment remains unclear. A mild ABP reduction might not effectively correct the luxury perfusion, while a rapid comprehensive therapy could increase the risk of ischemia, especially for patients with intracranial arterial stenosis ≥70% (64). Nazir et al. (65, 66) reduced the mean ABP by an average of 9.3 and 9.5 mmHg within 4–8 and 2–7 days after mild ischemic stroke event (NIHSS scored 0–5) in patients with normotension and mild hypertension (with mean ABP between 110 and 145 mmHg), respectively. No significant change occurred in ICA flow, MCA velocity or CBF measured by SPECT. It was noteworthy that patients with >70% stenosis or occlusion of carotid or vertebral arteries were excluded from these two studies (65, 66). In the study by Powers et al. (67), 11 subjects, with systolic BP higher than 145 mmHg, underwent a rapid reduction of mean ABP by 16 ± 7 mmHg 1–11 days after hemispheric ischemic stroke. However, two subjects showed CBF reductions of >19% in both hemispheres detected by PET, and one of the two suffered clinical deterioration with an increase of NIHSS scores from 1 to 5 (68). These findings demonstrated the capability of cerebral autoregulation after ischemic stroke varied from patient to patient, but the failure of autoregulation did occur in some individual.

For the patients who suffer hyperperfusion following full recanalization and without a stenosis ≥70%, maintaining systolic BP < 140 mmHg under careful observation might be safe (17, 69). Randomized control trials are warranted to examine whether antihypertensive therapy could improve the prognosis of patients with post-stroke hyperperfusion. If hyperperfusion was combined with severe intracranial stenosis, strict BP management might lead to neurological deterioration. We suggested evaluating cerebral autoregulation at bedside before deciding the ABP target if possible. A patient under neurocritical conditions could benefit from maintaining the ABP at the level when the cerebral autoregulatory function achieved the best (optimal ABP, ABPopt). Deviation of ABP from ABPopt was associated with cerebral perfusion worsening and unfavorable neurological outcomes (70–74). Cerebral autoregulation evaluation usually needs continuous TCD and ABP monitoring, to record the waveforms of bilateral MCA blood flow and arterial pressure. Data were analyzed by using specific software such as ICM+ software (University of Cambridge, Cambridge, United Kingdom). However, the devices and the software are not widely available in most stroke centers.

## Conclusions and Expectations

Hemodynamic recovery after recanalization brings about a better outcome in ischemic stroke. Both hypoperfusion and hyperperfusion may lead to cerebrovascular complications and poor outcome. With various modalities, assessment after recanalization may aid in early identification of patients with high risks and intervention in those with hemodynamic disturbances based on the evaluation to reduce the risk of complications and improve clinical outcome. Most of the available research studies are focused on cerebral perfusion and CBF velocity, and more studies are needed to concentrate on other modalities such as cerebral autoregulation and hemodynamic changes of the cerebral venous system (75). Furthermore, prospective studies are needed to confirm that hemodynamic management based on evaluation, particularly the cerebral autoregulation assessment, may improve the outcome of recanalization therapies. The cost-effectiveness of various hemodynamic evaluation modalities also needs to be analyzed.

## Author Contributions

LL: topic design. ZZ: searched literature and wrote this paper. YP and DM: revised the paper.

### Conflict of Interest Statement

The authors declare that the research was conducted in the absence of any commercial or financial relationships that could be construed as a potential conflict of interest.
